# Reduced EPO receptor expression may contribute to limited pleiotropic effects of EPO during critical illness

**DOI:** 10.1186/cc11047

**Published:** 2012-03-20

**Authors:** O McCook, S Matějková, J Matallo, A Scheuerle, P Moeller, M Georgieff, E Calzia, P Radermacher, H Schelzig

**Affiliations:** 1 University Medical School Ulm, Germany

## Introduction

We showed neuroprotective and renoprotective effects of recombinant human erythropoietin (rhEPO) after kidney and spinal cord ischemia/reperfusion (I/R) injury [1,2], but clinical studies using rhEPO to prevent acute kidney injury yielded equivocal results [3,4]. Increased cytokine release and/or oxidative stress can cause EPO resistance due to receptor modification and/or downregulation [5]. Since we recently failed to confirm rhEPO-related kidney protection in atherosclerotic swine [6], we compared kidney EPO receptor expression in swine strains with and without pre-existing vascular disease and kidney dysfunction.

## Methods

EPO receptor expression was quantified with immunohistochemistry (densitometric image analysis) of formalin-fixed paraffin sections from pre-injury kidney biopsies taken in young and healthy pigs (German Landrace, up to now *n *= 4) as well as swine (FBM strain, up to now *n *= 6) with familial hypercholesteremia (11.1 (7.4; 12.3) vs. 1.4 (1.3; 1.5) mmol/l, *P *< 0.001, *n *= 20 and 15, respectively, *P *< 0.001) and consecutive, diet-induced atherosclerosis [7].

## Results

Atherosclerotic swine presented with reduced glomerular filtration rate (creatinine clearance 76 (60; 83) vs. 103 (79; 120) ml/minute, *n *= 19 each, *P *= 0.004) and chronic histological kidney injury (dilatation of Bowman's space, swelling of Bowman's capsule, tubular dilatation and necrosis). EPO receptor expression was reduced by nearly two orders of magnitude in this strain (94.6 (8.3; 112.5)×10^7 ^vs. 1.7 (0.0; 4.7) ×10^7 ^densitometric units, *P *= 0.010).

## Conclusion

Even pretreatment with rhEPO did not influence I/R-induced acute kidney in swine with pre-existing impairment of kidney function and histological damage. The lacking beneficial effect of rhEPO was most likely due to the reduced expression of the EPO receptor, which may also explain contradictory results in clinical trials due to the frequent underlying kidney disease in the patients recruited.
